# Capturing the attentional response to clinical auditory alarms: An ERP study on priority pulses

**DOI:** 10.1371/journal.pone.0281680

**Published:** 2023-02-16

**Authors:** Vasco Ribeiro Ferreira, Ana Rita Pereira, Joana Vieira, Frederico Pereira, Rui Marques, Guilherme Campos, Adriana Sampaio, Alberto Crego

**Affiliations:** 1 Psychological Neuroscience Laboratory (PNL), Research Center in Psychology (CIPsi), School of Psychology, University of Minho, Braga, Portugal; 2 Sustainable Health Department, Campus Fryslân, University of Groningen, Leeuwarden, Netherlands; 3 Ergonomics & Human Factors Group, ALGORITMI Research Centre, University of Minho, Guimarães, Portugal; 4 CIAUD Research Centre for Architecture Urbanism and Design, Lisbon School of Architecture, Universidade de Lisboa, Lisboa, Portugal; 5 Center for Computer Graphics (CCG), Guimarães, Portugal; 6 Institute of Electronics and Telematics Engineering, University of Aveiro, Aveiro, Portugal; University of Southern Mississippi, UNITED STATES

## Abstract

Clinical auditory alarms are often found in hospital wards and operating rooms. In these environments, regular daily tasks can result in having a multitude of concurrent sounds (from staff and patients, building systems, carts, cleaning devices, and importantly, patient monitoring devices) which easily amount to a prevalent cacophony. The negative impact of this soundscape on staff and patients’ health and well-being, as well as in their performance, demand for accordingly designed sound alarms. The recently updated IEC60601-1-8 standard, in guidance for medical equipment auditory alarms, proposed a set of pointers to distinctly convey medium or high levels of priority (urgency). However, conveying priority without compromising other features, such as ease of learnability and detectability, is an ongoing challenge. Electroencephalography, a non-invasive technique for measuring the brain response to a given stimulus, suggests that certain Event-Related Potentials (ERPs) components such as the Mismatch Negativity (MMN) and P3a may be the key to uncovering how sounds are processed at the pre-attentional level and how they may capture our attention. In this study, the brain dynamics in response to the priority pulses of the updated IEC60601-1-8 standard was studied via ERPs (MMN and P3a), for a soundscape characterised by the repetition of a sound (generic SpO2 “beep”), usually present in operating and recovery rooms. Additional behavioural experiments assessed the behavioural response to these priority pulses. Results showed that the Medium Priority pulse elicits a larger MMN and P3a peak amplitude when compared to the High Priority pulse. This suggests that, at least for the applied soundscape, the Medium Priority pulse is more easily detected and attended at the neural level. Behavioural data supports this indication, showing significantly shorter reaction times for the Medium Priority pulse. The results pose the possibility that priority pointers of the updated IEC60601-1-8 standard may not be successfully conveying their intended priority levels, which may not only be due to design properties but also to the soundscape in which these clinical alarms are deployed. This study highlights the need for intervention in both hospital soundscapes and auditory alarm design settings.

## Introduction

Auditory warnings are pervasive in hospital wards and operating rooms, and considered key to patient safety. Ideally, these warnings should be detectable and learnable, resistant to auditory masking, but not annoying [1]. However, as increasingly recognised within the healthcare community, their sound design is generally poor, failing to meet the right compromise between those conflicting requirements [2, 3]. Added to the busy environment of hospitals, alarms are often loud and irritating, raising concerns about noise exposure of patients and staff. This problem has been listed as a top hazard in the medical community every year from 2012 to at least 2018 [4, 5]. The serious consequences of the noise in hospitals have shed light on the design of clinical auditory alarms, which should be urgently addressed.

Auditory alarms may become inaudible through auditory masking by other concurrent alarm sounds or noise sources sharing similar spectral content: Roy D. Patterson underlined this potential problem, proposing temporal and spectral design guidelines to avoid the masking phenomenon [6, 7]. Following Patterson’s pioneering work, a section on clinical alarm design guidelines was included in the early IEC60601-1-8 standard [8]. This standard and corresponding amendments were developed by IEC (International Electrotechnical Commission), with parts 1–8 of the document including general requirements for basic safety and essential performance regarding alarm systems incorporated in medical electrical equipment. Seeking to mitigate the identified issues, the standard proposes a set of alarm sounds to be used in clinical environments, defining eight alarm categories (e.g., oxygenation and ventilation), and including priority pointers for urgency discrimination (initially, Medium and High, with Low priority added in subsequent amendments). Based on simple note patterns designed by varying pitch, speed, and number of repetitions, the alarms followed a mnemonic rationale [9]. However, the set was not tested with real users at the time and there is increasing agreement that its design philosophy was flawed [2].

It has been pointed out that a method solely focused on melody and homogeneity between sounds is detrimental to learnability, with the intended mnemonic associations showing little to no effect in terms of reaction time (RT) or accuracy [3, 10, 11]. More importantly, the Medium and High Priority modifiers presented virtually no difference in the perception of urgency levels [12], a considerable hazard in an environment that demands quick action. Wee and Sanderson [2] showed that medical professionals had overall low performance not only in learning the set of sounds but also in recognizing them for their Medium or High Priorities, with only one participant out of twenty-two being able to achieve a 100% score on two consecutive trials during the entire experiment. Overall, these factors led researchers to conclude that the alarm pointers recommended in IEC60601-1-8:2006 were inefficient. Understanding the need to redesign the recommended alarm set, the IEC committee (which included not only acoustic and psychoacoustic researchers but also practitioners) began by establishing better methods to approach the task. The development of new alarms was, unlike the previous set, grounded on laboratorial and simulator user research.

**Fig 1:**
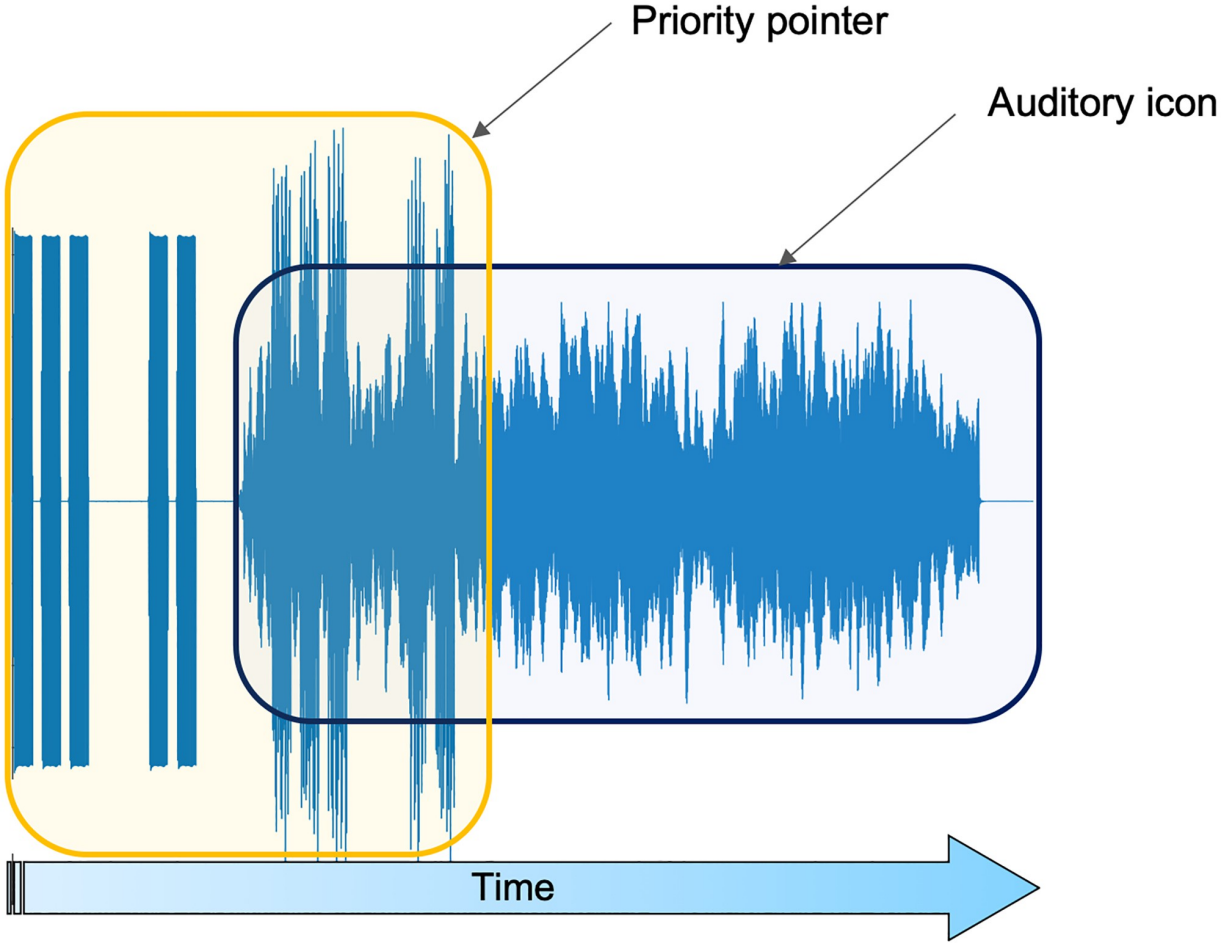
General structure of an alarm signal as recommended by the 2020 updated standard. The priority pointer is used to discriminate between the different urgency priorities (low, medium, and high). The auditory icon represents the alarm category.

In a subsequent 2020 update of the IEC60601-1-8 standard, the mnemonic system was replaced by auditory icons (see Fig 1): sounds from widely recognised everyday sources [13]. The eight alarm categories were now associated with familiar sounds. As an example, the Drug Administration sound consisted of pills rattling inside a plastic case, and the Cardiac sound resembled a heart beating. Strong indicators point to these as easier to learn, distinguish and locate [14]. However, adapting the icons and conveying priority without compromising their advantages are ongoing challenges, with priority pointers yet to be optimised to this day. The standard proposes as modulators of urgency to be used in the priority pointers sound design, acoustic parameters such as speed, rhythm, repetition, duration, pitch, and amplitude envelope.

Considering neuroscientific research on audiology, not much has been studied in terms of auditory alarms, and even less in the clinical alarm field. Electroencephalography (EEG) has been a widely used non-invasive technique in audiology research, reliable for measuring the electrophysiological brain response to a given stimulus. Event-Related Potentials (ERPs) register the brain response to a stimulus in a time span ranging from pre-attentive states to conscious recognition [15]. In this sense, ERPs allow for the observation of brain dynamics after stimulation, either visual or auditory, by displaying the waveforms of electrical activity that occur while processing specific information. With ERPs it becomes possible to identify and quantify neural processing, comparing measurable data such as the amplitude and latency of a component.

An early ERP component, the Mismatch Negativity (MMN) peaking between 150–200 ms around the Fronto-Central area of the brain, responds to unexpected sounds detected inattentively—a deviant sound, within a sequence of the same repeating sounds—a standard (generic) sound [16]. In this sense, exploring the MMN response to auditory alarms could offer relevant information on how these are perceived and detected against an environmental auditory scenario (e.g. operatory room), at the pre-attentional level when auditory processing occurs, according to the alarms’ different properties such as intensity and spectral components [17–19]. Likewise, a different ERP component, known as novelty P300 or P3a, may also be useful to assess the neural processing of these clinical alarms. Contrasting with the MMN, the P3a is a later (250–350 ms) positive component located at frontal regions that is intimately related to the attentional capture and focal attention and is usually elicited by an infrequent distracter stimulus inserted randomly into the target/standard sequence [20]. Thus, an infrequent distinct tone presented in a series of frequent tones without a task may elicit a P3a in frontal regions with a relatively short peak latency that habituates rapidly and is interpreted as reflecting frontal lobe activity [19]. Observing the P3a can be relevant to ascertain the identification and attentional capture of clinical alarms, providing detailed information pertaining to the allocation of resources needed to identify novelty auditory stimuli.

However, despite their potential to inform on how alarms are perceived and attended to, to our knowledge only one study used ERPs to test auditory alarms. The authors found that the repetition-suppression effect (habituation to sounds resulting in less effective attention) can be reduced by modulating pitch and intensity of alarms [21]. Complementary behavioural measures, such as RT (i.e., the interval between the onset of the stimulus and the initiation of response) and accuracy, may further support electrophysiological data: RT provides information regarding the attentional processing of the stimulus, by considering the time needed until a response is given [22].

The present study aims at implementing an electrophysiological approach to survey pointers from a recently developed auditory alarm set for medical electrical equipment. This approach relies on the ERP components examination to assess the Medium and High Priority pointers via their pulses, proposed in the updated IEC 60601-1-8 standard:2020.

Both priority pulses were tested within a sequence of auditory stimuli (i.e., a soundscape), composed by the repetition of a generic SpO2 beep (see Stimuli section), usually present in operating and recovery rooms. While reproducing each priority pulse overlaid to the beep sequence, we assessed its detection by scrutinising differences in amplitude for the MMN and P3a and the corresponding behavioural response. This study will provide a solid foundation for a better understanding of how these sounds are processed and how effective they are in capturing attention.

According to the design purpose of the tested priority pulses, it would be expected that at the neurophysiological level, the High Priority (HP) pulse corresponds to a larger amplitude for MMN and P3a, when compared to the Medium Priority (MP) pulse. Similarly, at the behavioural level, it is expected that the HP pulse will correspond to significantly shorter RTs and a higher response accuracy, than those from the MP pulse.

## Methods

### Participants

The experiments were conducted in accordance with the Declaration of Helsinki and received approval by the Institutional Ethics Committee for Social Sciences and Humanities of the University of Minho, Braga, Portugal (CEICSH 019/2020). All participants provided written-informed consent prior to the clinical interview, which was performed to assess the following exclusion criteria: use of illegal drugs; consumption of medical drugs with psychoactive effects (e.g., sedatives or anxiolytics) during the 2 weeks before the experiment; personal history of psychopathological disorders (according to DSM-V criteria); and history of traumatic brain injury, neurological disorder, or non-corrected sensory deficits.

#### Electrophysiological experiment

Participants were 29 healthy young adults (22 females) aged between 18 and 28 years (*M* = 20.86; *SD* = 2.77). Recruitment was done through an accreditation platform from the School of Psychology at the University of Minho, in which participants could attain extra credit for completing the experiment.

#### Behavioural experiment

36 healthy young adults (23 females) aged between 18 and 23 years and who did not partake in the electrophysiological phase were initially recruited for the second part of the experiment. Nine were excluded for not completing all the study trials, leading to a final sample of 27 (17 females) participants (*M* = 18.89, *SD* = .97). Recruitment was again done through an accreditation platform from the School of Psychology at the University of Minho.

### Stimuli

**Fig 2:**
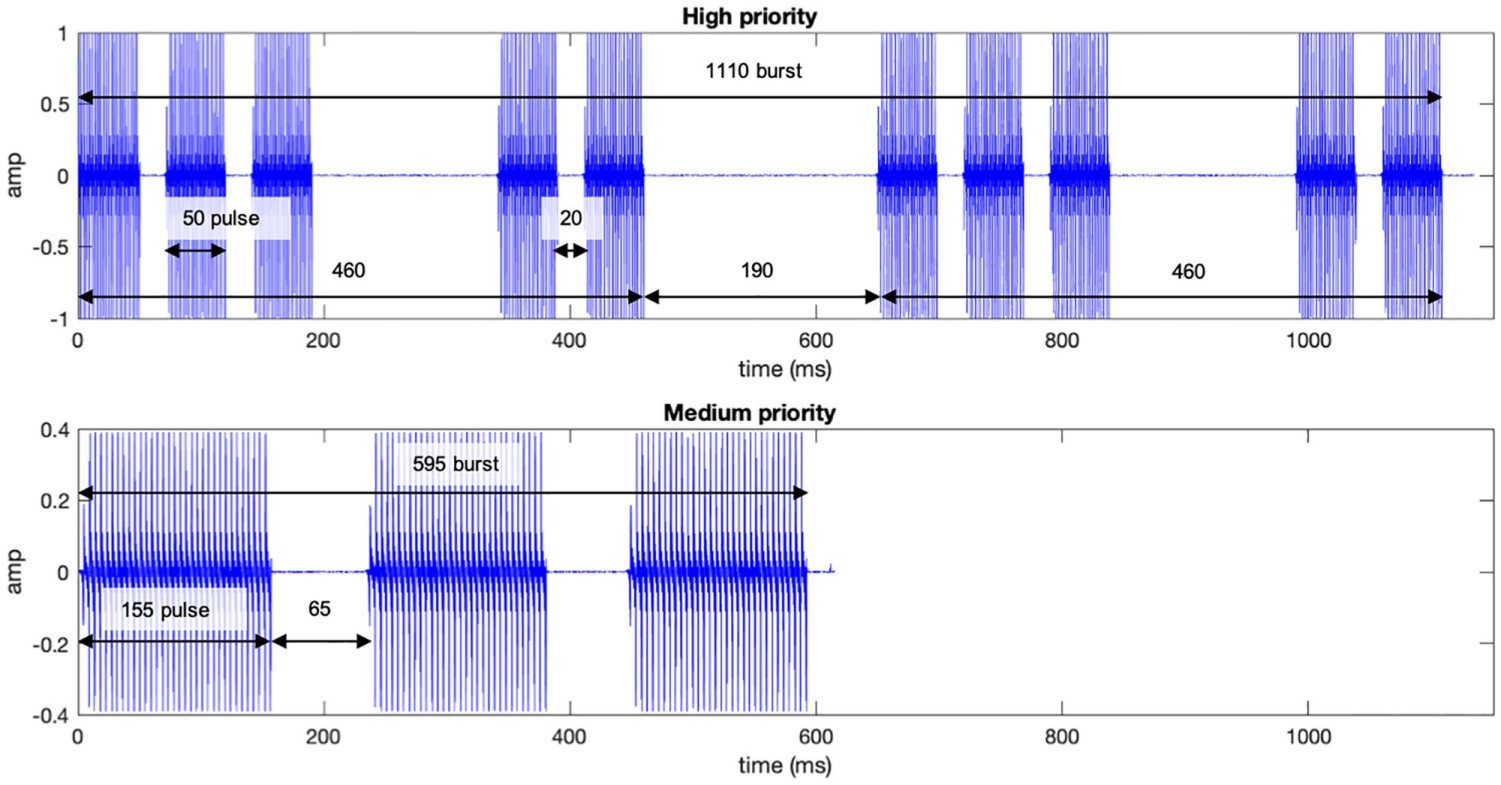
The HP pointer burst is composed of two identical five-pulse blocks separated by 190 ms, totaling 1110 ms of duration. HP pulses of 50 ms duration are repeated in the schematized rhythm pattern (top). The MP pointer burst has a total duration of 595 ms. MP pulses of 155 ms duration are repeated in the schematized rhythm pattern. The HP burst has a faster and uneven rhythm, features that are associated with a higher urgency perception.

The IEC 60601-1-8:2020 recommended priority pointers are constituted by *bursts*. A burst is a repetition of pulses arranged in a distinct temporal pattern. The priority pointer, i.e. a burst or a number of bursts, precedes an auditory icon that indicates the category of the source of the alarm condition. Four priority audio files were acquired from the IEC 60601-1-8:2020 supporting documentation (See S3 Data) and used as stimuli (see Fig 2). Two priority *burst* pointers, HP and MP, and the two correspondent *pulse* pointers.

**Fig 3:**
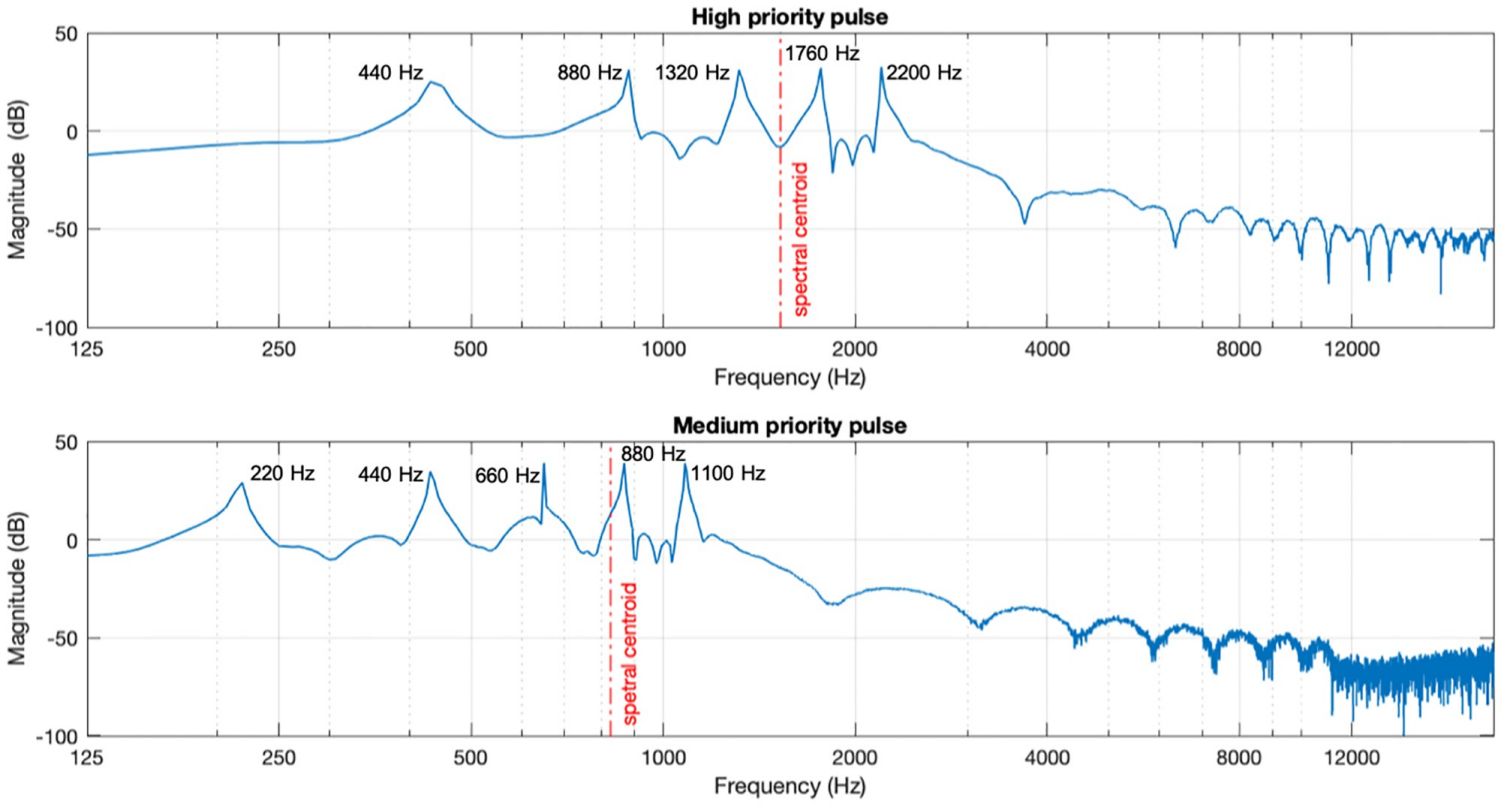
Spectral components of the HP (top) and MP (bottom) pulses. Spectral centroid frequency is represented by the vertical dashed line. Both pulses conform to the updated standard suggestion of having five peaks in the 150 Hz to 4000 Hz range, and with the HP signal being higher pitched than the MP.

The HP pulse shows a rise time and fall time of 5 ms, it has a spectral centroid of 1526 Hz. This signal has equally weighted components at 440 Hz, 880 Hz, 1320 Hz, 1760 Hz, and 2200 Hz for the first, second, third, fourth, and fifth harmonics, respectively. Correspondingly, these are the A4, A5, E6, A6, and C#7 musical notes, representing the A major chord in the chromatic scale. The MP pulse, with a rise time and fall time of 10 ms, has a spectral centroid of 825 Hz. This signal has equally weighted components at 220 Hz, 440 Hz, 660 Hz, 880 Hz, and 1100 Hz for the first, second, third, fourth and fifth harmonics, respectively. It consists of the musical notes A3, A4, E5, A5, and C#6, also making the A major chord, one octave below the HP. Both pulses are visually represented in Fig 3.

**Fig 4:**
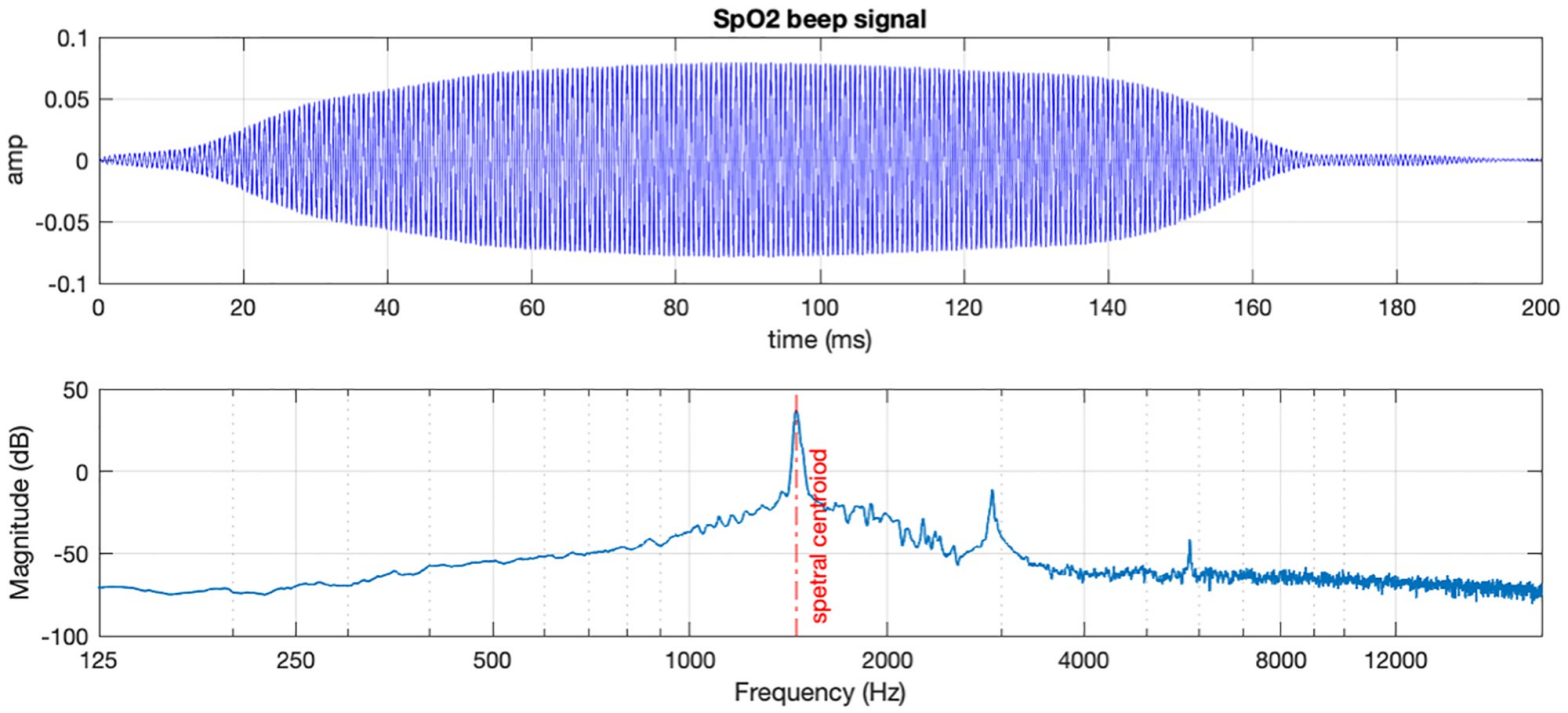
SpO2 generic beep recorded signal representation in time domain (top) and frequency domain (bottom). The 44100 Hz sample rate recording was done by one of the authors using a handheld recorder. The signal shows a fundamental at 1455 Hz, and two additional harmonic components of lower energy. Also visible are the longer rise and fall times than those of the priority pulses.

An additional stimulus, a generic SpO2 *beep*, a sound often present in hospital surgery and recovery rooms was used in the experimental tasks (see Fig 4). This signal was recorded from a vital signs monitor medical device. With a duration of 200 ms, the beep signal is essentially a tone with the fundamental at 1450 Hz, (showing two lower energy harmonic components), resulting in a calculated spectral centroid of 1450 Hz. Thus, the SpO2 beep differed from the priority pulses not only in the distribution of spectral components, but also in its duration and pattern of presentation (see Procedure).

The reproduction volume level for all sounds was adjusted to a comfortable listening level and kept the same for all participants. Each pulse signal used in the ERP and behavioural experiments has been pre equalised, so that estimates of Loudness [23] and Roughness [24] yielded for the High and Medium pulses a loudness of N5 = 24 sone, but with mean roughness of 0.9 asper and 0.5 asper respectively; for the SpO2 beep N5 = 18 sone and a very low roughness < 0.1 asper (estimates at listening levels of approximately 74 dB A for the three signals). On the other hand, for the burst pointers used in the behavioural experiment, estimated Loudness and Roughness levels were of N5 = 31 sone and mean roughness of 1.7 asper for HP; N5 = 16 sone, mean roughness of 0.6 asper for MP; and lastly N5 = 6 sone, with a similarly low mean roughness < 0.1 asper for the beep signal (estimates at listening levels of approximately 77 dB A, 70 dB A and 60 dB A respectively).

For the electrophysiological experiment, due to the temporal requirements of the experimental paradigm used to elicit MMN and taking into account the fact that the MMN begins at around 150 ms (with later processing corresponding to other components), tested sounds should have no more than 150 ms duration. For this experiment, the HP pulse (with 50 ms) and a shortened MP pulse (shortened to 150 ms) were used. For the behavioural experiment two conditions were created: (1) Using the HP and MP pulses (the same as those used in the electrophysiological experiment); and (2) using HP and MP burst pointers, presented at their full length (595 ms, and 1110 ms respectively).

In each of the experiments, priority pulses were compared in contrast to the generic SpO2 beep.

### Procedure

#### Electrophysiological experiment

Participants were instructed not to smoke, drink tea or coffee for at least three hours before the assessment. EEG data was collected inside the Psychological Neuroscience Lab, in the Electroencephalography section. After preparation and placement of electrodes, participants were seated in a comfortable armchair located in a light- and sound-attenuated electrically shielded room. Finally, a set of Sennheiser headphones were placed on their head and the EEG experiment was carried out. The same headphones and computer output volume settings were used throughout tasks and across participants.

The experiment involved two similar tasks programmed using Psychopy [25], namely the standalone version of PsychoPy 3.2.4 (https://github.com/psychopy/psychopy/releases/tag/3.2.4). The tasks are set out to elicit the MMN and P3a components, following an oddball paradigm, with one frequent generic tone (SpO2 beep) and one infrequent deviant tone (priority pulse) in each. There was no requirement for behavioural answers (i.e., the participants did not have to press any keys or identify the sounds). Participants were instructed to ignore the presented sounds, and instead lend their attention to a muted travel documentary video displayed during the course of the tasks. The two tasks were identical, differing only in the presented priority pulse (HP or MP) deviant stimuli. In both, the inter-stimulus interval (ISI) was variable between 0.5 s and 0.7 s, and, comprising 700 trials, each task had a total duration of 8 minutes. Each deviant stimulus had a probability of occurrence of 10% and the task design included a pseudo-randomized presentation of the stimuli, with the condition that all deviant stimuli had to be preceded by at least 4 generic stimuli, so to avoid presenting 2 consecutive or very closely spaced deviant stimuli.

The order of the tasks was counterbalanced across subjects. In addition, they were intercalated with a different 3-minute recognition task to avoid habituation, in a way that after performing the first task, they would have to perform an unrelated task, and only after that would they return to the second task.

#### Behavioural experiment

Participants were seated in a comfortable armchair located in a silent classroom. Although conducted in a different setting, the same set of headphones and comfortable volume adjustment as described above were used in this experiment. Two behavioural conditions were programmed and designed using Psychopy [25], namely the standalone version of PsychoPy 3.2.4 (https://github.com/psychopy/psychopy/releases/tag/3.2.4), to assess the recognition of priority pointers and the behavioural response to them. Both followed an oddball style in respect to HP and MP (target stimuli), with a generic SpO2 beep. After being familiarised with the two priority sounds and associating them to a key on the PC keyboard during a test phase, participants were required to press the corresponding key as soon as the priority pulses or pointers were heard. Instructions were given to ignore the generic SpO2 beep. The three sounds were presented sequentially and pseudo-randomly. The Inter-Stimulus Interval (ISI) was variable between 1 s and 1.5 s, with a total of 300 trials, each target had a probability of occurrence of 10%. The two conditions only differed in terms of the used target stimuli (i.e., pulses of a maximum 150 ms, or full length bursts). The order of the conditions was counterbalanced across subjects, with each having a duration of 12 minutes.

### EEG recording and processing

The EEG signal was recorded using the ActiveTwo Biosemi electrode equipment, following the 10–10 system [26] with 64 electrodes placed on the scalp. Continuous EEG signals were recorded referenced to a common mode sense (CMS) active electrode (placed between POz and PO4) and a driven right leg (DRL) passive electrode (located between POz and PO3), which together replace the classical “ground” electrodes. Additionally, electrooculogram (EOG) signals were monitored via two electrodes in the outer canthi of the eyes and another one below the left eye, in order to later correct EEG signals for ocular movements artifacts. Finally, one external electrode that served as an offline reference was placed on the tip of the nose (ToN). Preamplifiers in each electrode were used to lessen the induced noise between the electrode and the amplification/digitization system (BioSemi ActiveTwo, BioSemi B.V. Amsterdam), thus allowing high electrode impedances. Electrode offsets were kept between ± 30 mV, the EEG signal was amplified and digitised at 512 Hz rate and filtered online with a 0.01–100 Hz bandpass filter.

EEG data was processed and visualised using BrainVision Analyzer 2.1 (Brain Products GmbH, München, Germany). The signal was re-referenced to ToN, and then filtered digitally off-line between 0.1 and 30 Hz via phase shift free Butterworth filter (24 dB/octave roll-off) with an additional notch filter at 50 Hz. The EEG was corrected for ocular artefacts by the semi-automatic procedure in Independent Component Analysis (ICA) [27] as implemented in BrainVision Analyzer. EEG was segmented into epochs from 100 ms prior to stimulus onset to 500 ms after. Prestimulus baseline was adjusted to 0 μV, and epochs with artifacts exceeding ±80 μV at any datapoint were rejected. Then, in order to obtain the ERPs, epochs were averaged according to the different stimulus (standard and deviant) separately for each subject, excluding from the analysis the standard stimuli presented immediately after the deviant stimuli.

Finally, in order to calculate the MMN and P3a, the “difference waveforms” were obtained by subtracting from each subject separately the ERPs elicited to standard stimuli from deviant stimuli in the corresponding category (Medium and High priority).

### Principal component analysis

**Fig 5:**
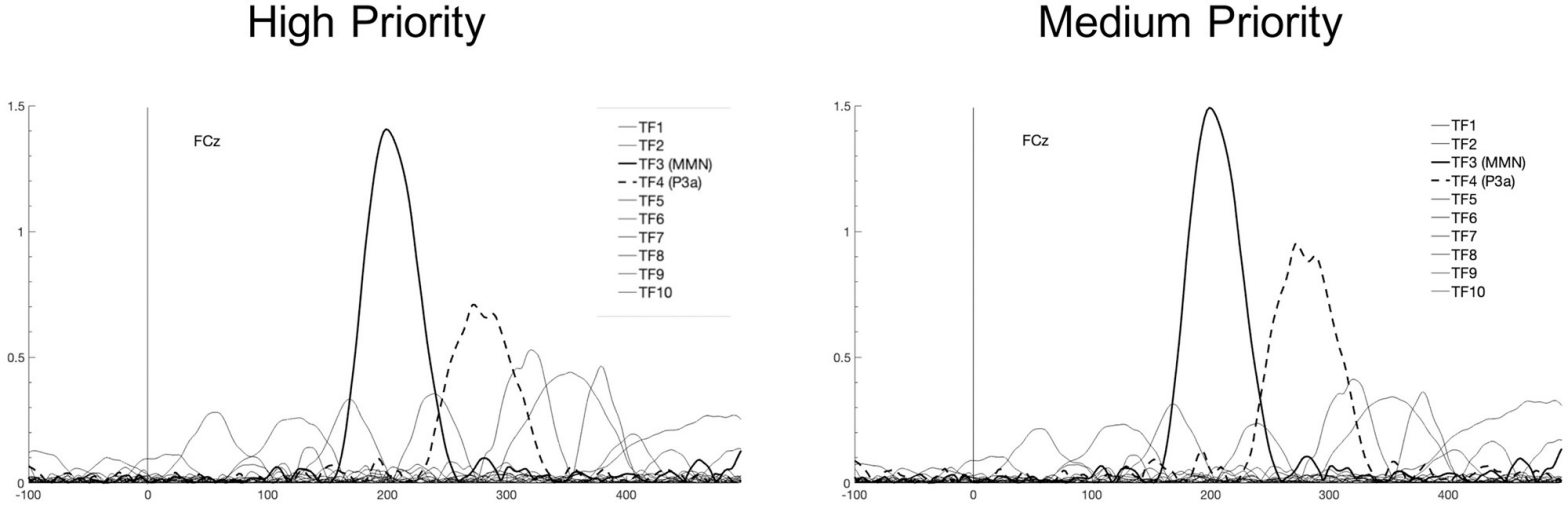
Plots of the Grand average of the waveforms of factor (component) loadings in response to each priority at the location of the maximum peak (FCz). Temporal factors identified as MMN and P3a are highlighted in bold solid and dashed lines respectively.

“Difference waveforms” for each category (Medium and High priorities) were exported from BrainVision Analyzer and then examined by principal component analysis (PCA) with Dien’s ERP PCA toolkit (v. 2.68) [28] in MATLAB (The Mathworks, v. 9.4.0.885841, R2018a, Natick, MA, USA). Data was resampled to 250 points/s. Temporal principal component analysis was then computed with promax rotation. Parallel testing [29] resulted in selection of 10 factors with a total variance of 0,7479 (see Table 1 and Fig 5), of which the peak latencies, polarity, and explained variance are summarised in Table 1.

**Table 1:** Temporal PCA factors: All the factors identified are ordered by the explained variance, with their latency (ms), maximum negative and positive locations and peak polarity. The factors with the event-related potential (ERP) components of interest for the study (MMN and P3a) are included in the final column.

Factor	Peak Latency (ms)	Peak (-) Channel	Peak (+) Channel	Peak Polarity	Variance	Unique Variance	ERP Wave Identified
TF1	489.8438	Iz	T7	+	0.2626	0.1145	
TF2	353.125	Iz	Cz	+	0.1515	0.0618	
**TF3**	198.8281	FCz	P9	-	0.1515	0.0973	**MMN**
**TF4**	273.0469	P10	FCz	+	0.1426	0.0695	**P3a**
TF5	126.5625	F7	C2	+	0.0610	0.0314	
TF6	405.8594	Pz	C5	+	0.0322	0.0214	
TF7	167.5781	FC4	P10	+	0.0299	0.0231	
TF8	319.9219	P7	FCz	+	0.0243	0.0175	
TF9	239.8438	FC6	Iz	+	0.0235	0.0170	
TF10	56.25	F1	P8	+	0.0203	0.0145	

**Fig 6:**
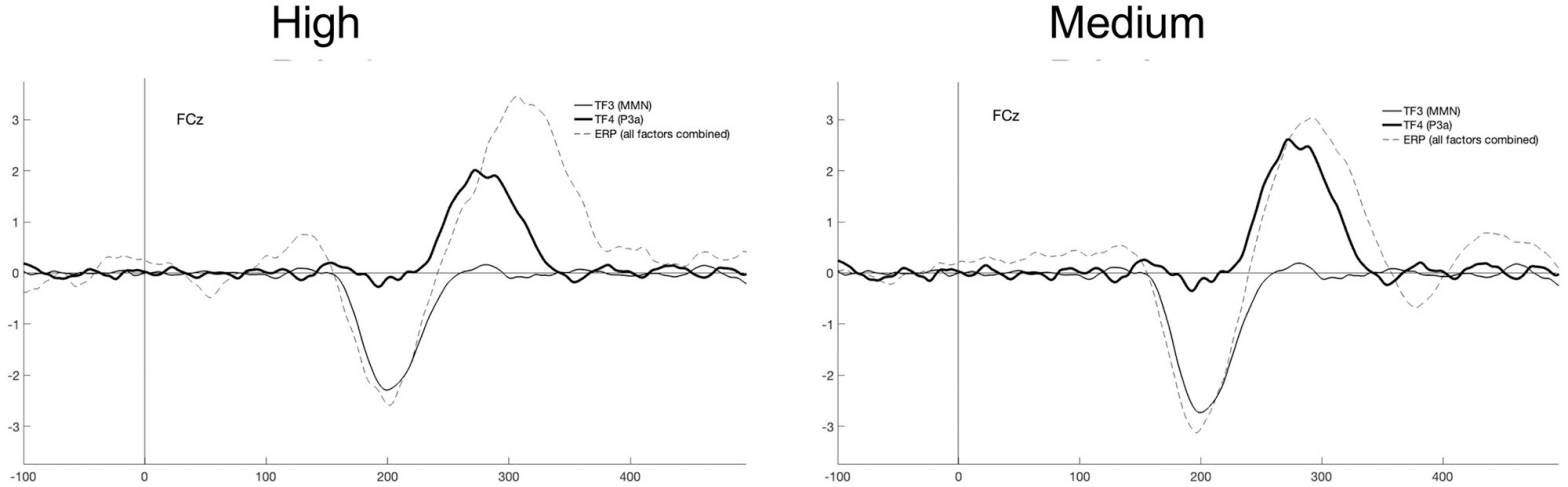
Plots of the Grand average of ERP (dashed line) versus Temporal Factor 3 and 4 of PCA identified as MMN (regular solid line) and P3a (bold solid line) respectively, for each priority at the location of the maximum peak (FCz).

The components extracted (MMN and P3a) were plotted as a time-course waveform rescaled to microvolts by multiplying the correlation factor loadings by the standard deviations of the variables to produce covariance loadings [30]. The factors and their plots were examined to identify corresponding ERP waves, which are illustrated in Fig 6.

The factor values were rescaled to μV and used for further analysis. The minimum negative peak at a 130–250 ms time window of TF3 (MMN) was extracted, for each subject and condition (Medium and High priorities) for six electrode locations (at the site of maximum amplitude and the central surrounding channels): three for Fronto-central Region (FC1, FCz, FC2) and three for Central Region (C1, Cz, C2). Likewise, the maximum positive peak at a 250–350 ms time window of TF4 (P3a) was extracted, for each subject and condition (Medium and High priorities) for nine electrode locations (at the site of maximum amplitude and the frontal surrounding channels): three for Fronto-Central Region (FC1, FCz, FC2), three for Frontal Region (F1, Fz, F2) and three for Antero-Frontal Region (AF3, AFz, AF4). Considering that the P3a has a wider scalp distribution, with attention modulation occurring at later stages of the P3a and leading to a more anterior distribution, we opted to include the anterior regions in this analysis [31]; in addition, the P3a has also been described as being modulated by the complexity and novelty of the deviant auditory stimuli being presented, with frontal activation increasing with task difficulty [32].

### Statistical analysis

Both ERP and behavioural data were statistically analysed (IBM Statistical Package for Social Sciences (SPSS), v.23). Regarding the ERP data, after verifying the assumptions of parametric tests, a repeated measures ANOVA with 2x2x3 design was used for MMN peak amplitude values with 3 Within-Subjects factors: Priority (with 2 levels)—Medium and High, Region (with 2 levels)–Fronto-Central and Central, and Electrode (with 3 levels)–l, z, 2 locations. Similarly, a repeated measures ANOVA with 2x3x3 design was used for the analysis of P3a peak amplitude values with 3 Within-Subjects factors: Priority (with 2 levels)—Medium and High, Region (with 3 levels)–Frontal, Fronto-Central and Antero-Frontal, and Electrode (with 3 levels)—l, z, r locations.

An alpha level of 0.05 was used and, whenever appropriate, degrees of freedom were corrected by the conservative Greenhousee Geisser estimate. All post hoc paired comparisons were performed with the Bonferroni adjustment for multiple comparisons, also with an alpha level of 0.05.

Behavioural performance was analysed across two variables related to RT (ms) and accuracy rates (correct responses) were calculated and registered by Psychopy software for each target (HP and MP) in both conditions. After not verifying the assumptions of parametric tests for all variables, the Wilcoxon signed rank test was used. Data will be presented according to task conditions (i.e., short, and full length).

## Results

### Electrophysiological results

**Fig 7:**
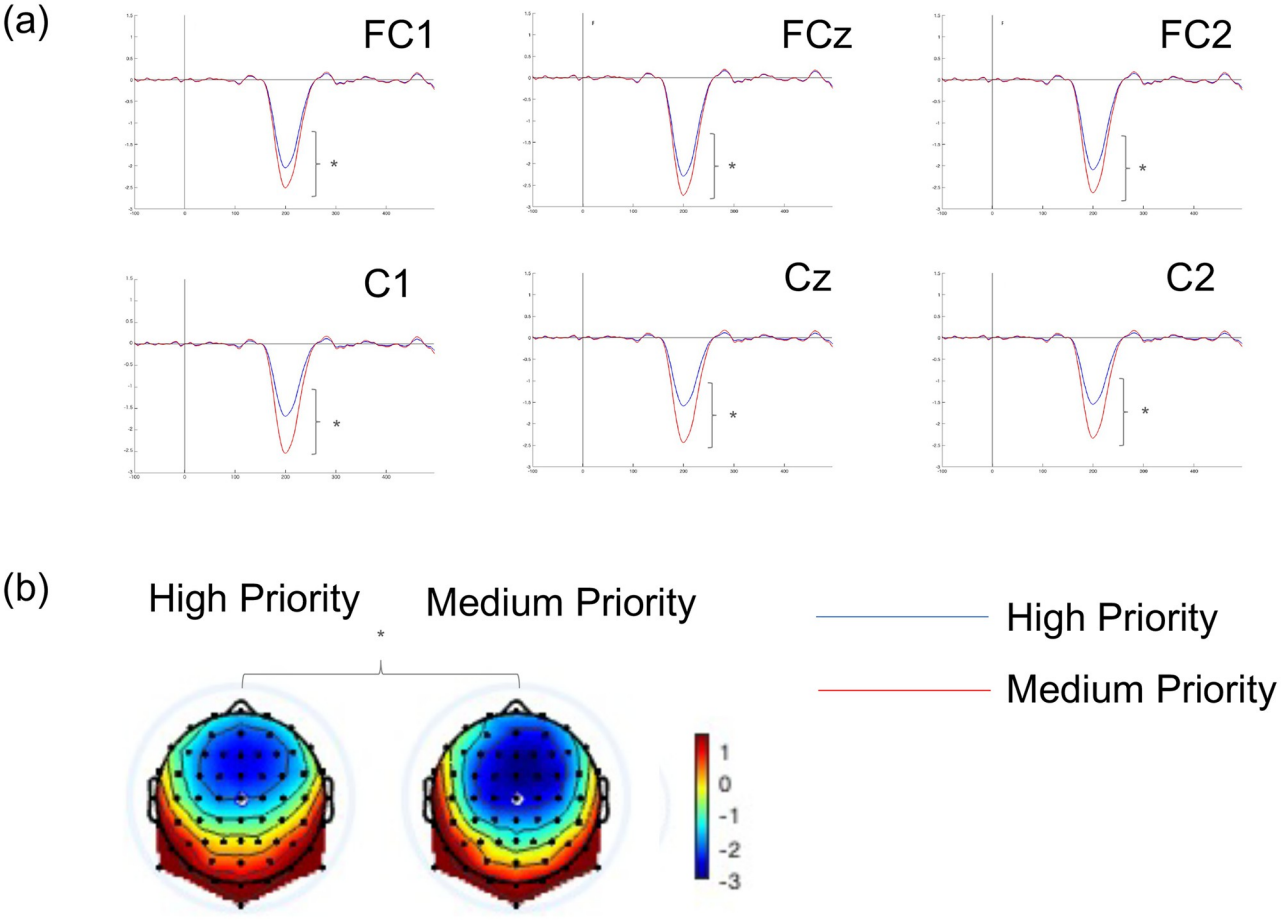
(a) MMN (TF03) waves for high priority (blue lines) and medium priority (red lines) pulses of the fronto-central and central regions; and (b) voltage topographical distributions to the peak of the MMN (198 ms) in response to the high priority and medium priority pulses (* p < .05).

The analysis of MMN revealed that Priority had a significant effect [F(1, 28) = 4.78, *p* = .04]. The MMN peak amplitude was significantly larger for the Medium (*M* = -2.49, *SD* = .34) than in the High (*M* = -1.79, *SD* = .22) category (see Fig 7). Moreover, there was also a significant effect for Region [F(1, 28) = 4.69, *p* = .04]. The MMN amplitudes were significantly larger for the Fronto-Central (*M* = -2.25, *SD* = .26) than Central (*M* = -2.03, *SD* = .22) region. No significant differences were observed in relation to interactions between factors.

**Fig 8:**
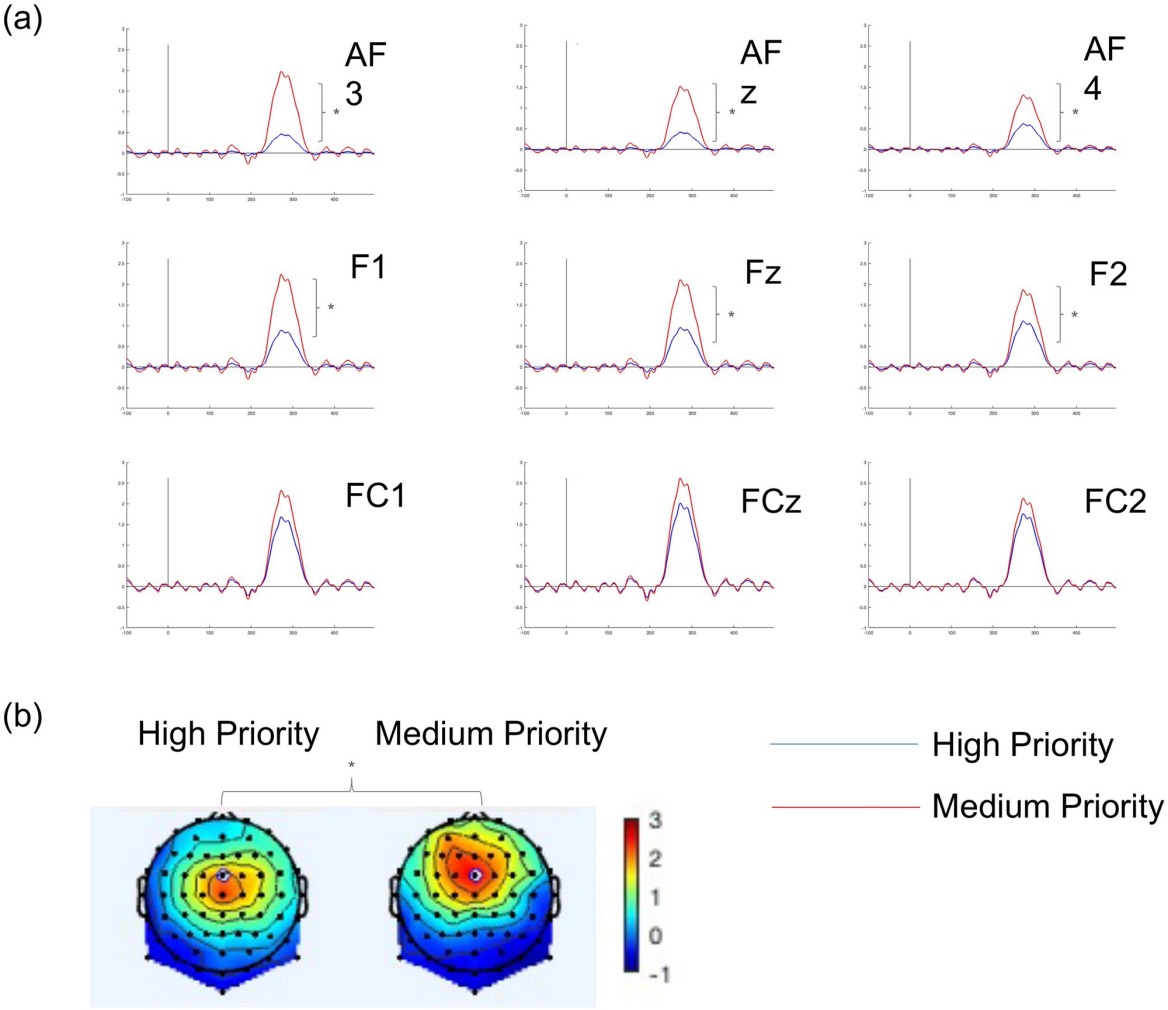
(a) P3a (TF04) waves for high priority (blue lines) and medium priority (red lines) pulses of the Antero-Frontal, Frontal and Fronto-Central regions; and (b) voltage topographical distributions to the peak of the P3a (273 ms) in response to the High priority and Medium priority pulses (** p < .01).

With regard to P3a, the analysis revealed that Priority had a significant effect [F(1, 28) = 6.38, *p* = .02]. The P3a peak amplitude was significantly larger for the Medium (*M* = 2.02, *SD* = .32) than in the High (*M* = 1.12, *SD* = .31) category (see Fig 8). Moreover, there was also a significant effect for Region [F(2, 56) = 40.24, *p* < .01]. The P3a amplitudes were significantly larger for the Fronto-Central (*M* = 2.09, *SD* = .27) than Frontal (*M* = 1.54, *SD* = .28) and Antero-Frontal (*M* = 1.07, *SD* = .25) region. The analysis also revealed significant interactions between Priority and Region [F(2, 56) = 4.75, *p* = .01]. The post hoc multiple comparisons (adjusted by Bonferroni correction) showed that P3a peak amplitude was significantly larger for the Medium than the High priority in Antero-Frontal (*p* < .01) and Frontal (*p* < .01) regions, whereas there were no significant differences in Fronto-Central region (p = .20) (see Fig 8).

### Behavioural results

Data for each category (HP and MP) in each condition (short and full length) are summarised in Table 2.

**Table 2:** RT and accuracy behavioural data by condition (short length and full length).

	High Priority Md (range)	Medium Priority Md (range)	*Z*	*p*	*r*
**RT—Short length**	674 (707)	611 (558)	- 4.06	< 0.001***	-.78
**RT—Full length**	700 (697)	664 (674)	-2.04	.04*	-.39
**Acc—Short length**	.85 (.45)	.94 (.52)	-1.79	.075	-.34
**Acc—Full length**	.94 (.52)	.94 (.58)	-.16	.882	-.03

Acc, accuracy; Md, median; RT, reaction time (ms);

* *p* < .05;

*** *p* < 0.001.

For RT, in the short length condition (pulse pointers of short duration), results revealed that RT for the MP pulse (*Md* = 611 ms, *n* = 27) were significantly shorter when compared to the HP pulse (*Md* = 674 ms, *n* = 27), *z* = -4.06, *p* < 0.001, with a large effect size, *r* = -.78. In the full length condition (full length burst pointers), results also revealed that RTs for the MP pointer (*Md* = 664 ms, *n* = 27) were significantly shorter when compared to the HP pointer (*Md* = 700 ms, *n* = 27), *z* = -2.04, *p* = .041, with a medium effect size, *r* = -.39.

When assessing accuracy, no significant differences were observed in the short or full length condition between MP and HP pointers.

## Discussion

The present study aimed to observe the attentional response to clinical auditory alarms. ERPs were recorded while participants were exposed to the priority and beep sounds inattentively. Additionally, in subsequent behavioural experiments, participants were instructed to attentively respond accurately and quickly to the heard priority sounds.

Contrary to our hypothesis, electrophysiological results showed larger amplitudes of the MMN and P3a for MP pulses, when compared with the HP pulses, suggesting higher detection (at the pre-attentive level) of a change of acoustic environment and attentional capture at the neural level for MP pulses. Likewise, behavioural results were contrary to our hypotheses and, in line with electrophysiological results, they point to faster conscious processing for MP, supported by shorter RT in comparison to HP.

The auditory MMN appears whenever a deviant sound is detected inside a sequence of repeating identical sounds. With the MMN amplitude increasing with the difference between deviant and standard sounds, higher values for MMN have been interpreted as a larger electrophysiological response in relation to a given sound [33]. Translated into the context of auditory processing, unique mechanisms appear to be involved in the automatic detection of sounds: a “change-detector”, observed by presenting a distractor, results in the activation of the distraction potential (i.e., a brain network of involuntary attention) [19].

The electrophysiological data of the P3a component appears to be in accordance with MMN findings that MP pulses are being easily processed when compared to the HP pulses. The differences in amplitude in the P3a are not given by sensorial detection of a sound, as seen in the MMN, but by directing attention to that sound. While the MMN is the manifestation of an automatic process, the P3a can be considered as a product of complex top-down mechanisms that guide and shift attention [19]. In this sense, a larger amplitude for the MP pulses may be the product of a larger activation of the distraction potential network, when compared to HP. In light of P3a findings, it is evident that an effective shift in attention towards the priority pulses took place, and that this attention change is more pronounced when a MP pulse is detected as opposed to HP. Amplitude changes in the P3a are modulated by the stimulus context, as stated previously, varying according to the properties of the deviant sound. Thus, higher amplitudes have been previously linked with a higher allocation of attentional resources.

In the case of the present study, a larger peak amplitude of MMN and P3a for one of the sounds translates into a higher neural early response to that sound, in contrast to the other. Since the MMN and P3a response are influenced or modulated by the perceptive difference in relation to a given standard stimulus, it is not possible to conclude with certainty that this difference in the response is not tied to possible acoustic similarities between stimuli used (i.e., deviant and standard). However, as demonstrated by a subsequent behavioural experiment, a shorter RT for MP pulse appears to support the electrophysiological data implications, even though there was no significant effect in accuracy. Although higher accuracy for HP was expected, finding no significant differences for accuracy may be explained by the fact that MP and HP pulses may differ enough in terms of acoustic properties, and are therefore easily distinguishable between them, even though not as easily detected as suggested by RT findings.

Previous behavioural studies appear to point towards an opposite trend, in which HP warnings have been processed faster when compared to the Medium and Low Priority [34, 35]. Furthermore, a previous study suggested that shorter pulses are often perceived as more urgent, as evidenced by manipulating pulse interval and alert duty cycle, which was surprisingly uncorroborated by our findings (i.e. a shorter pulse for HP was expected to be quickly attended) [36]. However, it has already been suggested that Priority pointers are not conveying priority as expected, with performance being faster and more accurate for MP [2]. Indeed, there is a vast body of literature [37–42] indicating acoustic requirements such as high frequency, shorter inter-pulse intervals or pulse length to better communicate priority—all accounted for in the current IEC60601-1-8:2020 standard. Additionally, the updated standard’s priority pointers have been designed to better resist masking.

The priority mapping has been mostly validated via models and laboratorial [43] or simulated [44] behavioural tasks, though very little is known of their use in the real world. Our results could highlight the fact that the current priority pulses, and consequently the pointers in the updated IEC60601-1-8:2020 standard are still yet to be optimised. Given the exploratory aspect of this study, at the moment these results should be replicated by future research and more investigation is necessary, but researchers should take further advantage of the auditory ERPs to study clinical auditory alarms, not only by observing how they are processed against different soundscapes, but also by manipulating different acoustic properties of the warnings themselves. This experiment underlines the relevance of studying the electrophysiological brain response to clinical auditory alarms. At the electrophysiological level, this study delves deeper into how alarm sounds are processed both at an automatic level with the MMN, and at an attention-oriented level with the P3a. The scarcity of studies focusing on the way we process these alarms reveal a gap in the knowledge regarding their optimisation. Taking into account the convergent findings between behavioural and electrophysiological data, it is possible to ascertain EEG as a reliable methodology to assess the automatic attentional response to clinical alarms, added to the fact that the latter ultimately provides additional relevant information to this processing, allowing for an accurate quantification. To the present time, and based on this brain response measure, we still cannot be certain that the clinical auditory alarms available in the updated IEC60601-1-8:2020 standard are successfully conveying their relative priority levels. However, it is fair to conclude that these alarms’ design could still yet benefit from a deeper understanding of their neural processing and how they are processed in relation to different soundscape conditions, namely the operating or recovery rooms in which clinical alarms take place.

This study also raises some limitations. First, it is important to highlight that the generic SpO2 beep used in the electrophysiological experiment may share acoustic properties with the priority pulses. These similarities, such as pitch, may lead to one of the pointers resembling the beep more than the other. In a real-life context, it might be possible that typical background noises in hospitals interact with the detection of the Priority pointers, potentially reducing their effectiveness. Another limitations are the small sample size and the characteristics of the sample included in this study; taking into account the younger representation due to most participants being students, not representative of the hospital population, it may be important to point out that perhaps they did not differ much between them, not allowing for a more diverse sample and consequently compromising generalisation.

In conclusion, the results of this study indicate that the current priority auditory warnings may not be conveying their intended urgency levels discrimination. This conclusion is evidenced by a larger automatic brain response and faster behavioural conscious response for the MP pulses than for the HP pointers of the updated IEC60601-1-8:2020. Additionally, these results suggest a need to intervene and improve not only clinical alarms but also possibly the soundscapes themselves, ultimately optimising the work environment, focusing on safety, effectiveness, and pleasantness.

## Supporting information

S1 Data File concerning all ERP data analysed in this article. (SAV)

S2 Data File concerning all behavioural data analysed in this article. (SAV)

S3 Data URL for the IEC 60601-1-8 standard sources. (PDF)
