# Correction: Capturing the attentional response to clinical auditory alarms: An ERP study on priority pulses

**DOI:** 10.1371/journal.pone.0295826

**Published:** 2023-12-07

**Authors:** Vasco Ribeiro Ferreira, Ana Rita Pereira, Joana Vieira, Frederico Pereira, Rui Marques, Guilherme Campos, Adriana Sampaio, Alberto Crego

The last author, Alberto Crego, is incorrectly noted as the corresponding author. The correct corresponding author is Guilherme Campos. Guilherme Campos’s email address is: guilherme.campos@ua.pt.

Three additional affiliations are missing for the sixth author. Guilherme Campos is also affiliated with the Institute of Electronics and Informatics Engineering of Aveiro (IEETA), University of Aveiro, Portugal; Department of Electronics, Telecommunications and Informatics (DETI), University of Aveiro, Portugal and Intelligent Systems Associate Laboratory (LASI), Portugal.

The following information is missing from the Funding statement: This work was also partially funded by FCT—Fundação para a Ciência e a Tecnologia (FCT) I.P., through national funds, within the scope of the UIDB/00127/2020 project (IEETA/UA, http://www.ieeta.pt/). The authors express their gratitude for this support.
