# Supplementary material for: Capturing the attentional response to clinical auditory alarms: An ERP study on priority pulses
Source: PLoS One. 2023 Feb 16;18(2):e0281680. doi: 10.1371/journal.pone.0281680 (PMC9934403; doi:10.1371/journal.pone.0281680)
Supplement: S3 Data — (PDF) [file pone.0281680.s003.pdf]

S3 URL for the IEC 60601-1-8 Standard sources:

https://webstore.iec.ch/publication/59648
